# Helicobacter pylori infection in rheumatic diseases

**DOI:** 10.1186/ar3675

**Published:** 2012-02-09

**Authors:** Hongyan Wen, Jing Luo, Junxia Li, Xiaofeng Li

**Affiliations:** 1 Department of Rheumatology, Shanxi Medical University, The Second Hospital of Shanxi Medical University, 56 South Xinjian Road, Taiyuan, Shanxi Province, China

## Background

Due to a number of factors, Helicobacter pylori (Hp) infection is increasingly recognized as highly prevalent in many populations and of increasing health concern. Hp infection has been associated with digestive diseases and rheumatic diseases[1]. It remains unclear whether all or part patients of rheumatic diseases should be routinely screened for Hp infection. We have examined predictors of Hp infection in rheumatic diseases so as to define who might benefit most from screening.

## Methods

292 patients with rheumatic diseases were recruited through outpatient rheumatology clinics between 2005-2008. The study was approved by the Second Hospital of Shanxi Medical University Ethics Committees, and all participating patients signed an informed consent form. The description of this study is 3-fold: to evaluate the relationship between Hp and rheumatic diseases, to assess the relationship between Hp and rheumatoid arthritis (RA), to explore the relationship between Hp and ankylosing spondylitis (AS).

## Results

Patients of rheumatic diseases were significantly more likely to be Hp infection than health control (89 vs 42%, P < 0.01). The study revealed that 88% of RA patients and 90% AS patients suffer from Hp infection. RA patients carried a diagnosis of Hp, a higher prevalence of the value of CRP was associated with the DAS28(Disease Activity Scor-28) (r = 0.287,P = 0.034). AS patients carried a diagnosis of Hp, a higher prevalence of the value of MMP-3(matrix metalloproteinase-3, MMP-3) was associated with the BASDI(Bath AS Disease Activity Index) (r = 0.435,P = 0.009).

## Conclusions

Patients of RA and AS are associated with a high prevalence of Hp infection rate. Hp infection may be play an important role in RA and AS.

## Next steps

Further investigation with other rheumatic diseases are planned.
